# Increased expression of neuregulin 1 in the urothelium of rat bladder with partial bladder outlet obstruction

**DOI:** 10.1186/s12894-017-0307-2

**Published:** 2017-12-08

**Authors:** Seung Woo Yang, Seong Woo Jeong, Ki Hak Song

**Affiliations:** 1 Department of Urology, School of Medicine, Chungnam National University Hospital, Chungnam National University, 282 Monwha-ro, Jung-gu, Daejeon, Republic of Korea 35015; 2 Department of Physiology, Yonsei University Wonju College of Medicine, Wonju, Republic of Korea

**Keywords:** Neuregulin-1, Receptor, ErbB-2, Outlet obstruction, Bladder, Rats

## Abstract

**Background:**

This study determined whether changes in the expression of neuregulin (NRG) 1, erbB2 tyrosine kinase (ErbB2) and the M2 muscarinic receptor in the urothelium and detrusor muscle of the rat bladder were associated with partial bladder outlet obstruction (PBOO).

**Methods:**

Male Sprague-Dawley rats (body weight 250–300 g) were used and subdivided into control (*n* = 10) and PBOO groups (*n* = 20). PBOO was induced for 21 days, and the expression of NRG1, ErbB2 and M2 muscarinic receptor mRNA and protein was evaluated using reverse transcriptase-polymerase chain reaction (RT-PCR) and western blotting, respectively.

**Results:**

In the urothelium of rat bladder samples, protein expression and mRNA expression of NRG1, ErbB2 and M2 muscarinic receptor were significantly increased in the PBOO group compared to the control group (*p* < 0.05). Only mRNA expression levels of NRG1/ ErbB2 were higher in the detrusor muscle of the PBOO group compared to the control group (*p* < 0.05).

**Conclusions:**

Our study demonstrated remarkable changes in the expression of NRG1/ErbB2 receptor mRNA and protein in the urothelium and muscle layer. These results suggest that NRG1 overexpression plays some kind of role against the PBOO-induced upregulated muscarinic receptors in detrusor overactivity.

## Background

Bladder outlet obstruction (BOO) is caused by common disorders that result from congenital anomalies, such as bladder neck sclerosis, posterior urethral valve, urethral stricture and benign prostatic hyperplasia (BPH) [1, 2]. BOO may lead to detrusor overactivity via cholinergic denervation of the detrusor muscle and subsequent acetylcholine hypersensitivity [3]. Overactive bladder (OAB) induced by partial BOO (PBOO) is derived from an aberration in the micturition reflex, which leads to involuntary detrusor contraction of either myogenic or neurologic origin [4]. Several studies demonstrated that the urothelium of bladder is more than a simple barrier because it actively contributes to bladder function [4–7]. The urothelium may play a role in the mechanosensory transduction during voiding via its ability to release many substances in response to stretch during filling, including ATP [8, 9], prostaglandins [9], nitric oxide [10], acetylcholine [11] and nerve growth factor [12], which affect nerve, smooth muscle, interstitial and immune cells.

Neuregulin (NRG) 1 is a trophic factor in the family of growth factors, and it contains an epidermal growth factor (EGF)-like domain. The roles of NRG1 in synaptic plasticity, neuronal and glial development and cardiomyocyte regeneration after heart injury were investigated extensively [13, 14]. Multiple binding profiles and various G-coupled protein receptors modulate NRG1/ErbB signalling, and we reported previously that NRG1/ErbB signalling was associated with inflammatory states of the rat bladder, such as interstitial cystitis [15]. NRG1-mediated ErbB activation modulates interneuron function. However, the roles of NRG1/ErbB signalling in the regulation of PBOO-induced OAB are not known.

The present study evaluated changes in the expression and distribution of NRG1/ErbB2 and M2 muscarinic receptors in the urothelium and detrusor muscle of rat bladders subjected to PBOO and determined whether these changes were associated with PBOO-induced OAB.

## Methods

### Experimental animals and PBOO surgery

Male Sprague-Dawley rats (250–300 g, Daehan Biolink Co. Ltd., Daejeon, Korea) were used in this study, and all experiment animals followed a protocol approved by the Ethics Committee on Animal Research at Chungnam National University, Daejeon, Korea (CNU-00261). Animals were raised in pairs in a cage covered with sawdust. Water and food were provided ad libitum. The temperature was controlled at 25 °C ± 1 °C, and the humidity was maintained at 55% ± 5%. Illumination varied in 12-h intervals. Thirty rats were used in this study, and rats were randomly divided into two groups (20 in the PBOO group and 10 in the control group). Each rat was anaesthetized using intramuscular ketamine (15 mg/kg) and rompun (5 mg/kg). A lower midline abdominal incision was made and deepened into the pelvic cavity to expose the bladder and urethra. A 3–0 silk ligature was placed around the urethra, and a 24-gage angio needle sheath was used to create a partial BOO in the PBOO group. The sheath was removed, and the incision was closed. PBOO was created using previously reported methods [16]. A sham operation was performed in the control group under same circumstances as the PBOO group.

### Tissue preparation for analyses

Rats were anaesthetized 21 days after PBOO induction in the same manner described above, and cystectomy was performed through an abdominal incision at the level of the ureteric orifices. The rats were sacrificed by carbon dioxide inhalation. Specimens were divided vertically into two portions (one half for RT-PCR and one half for western blotting). The divided bladder tissue was placed into a dish containing a phosphate-buffered saline solution. The urothelium and detrusor muscle were separated using microscissors and microforceps under the direct vision of a phase-contrast microscope.

### Total RNA extraction

Total RNA was extracted from the urothelium and detrusor muscle of control and PBOO rats. Tissue was homogenized in a 5-mL glass tube containing 0.8 mL of TRIzol (Invitrogen, Carlsbad, CA, USA) for 5 min at room temperature. The homogenate was transferred to a 1-mL tube, mixed with 160 μL of chloroform for 5 min at 4 °C, and centrifuged for 10 min at 13,200×g at 4 °C. The supernatant was transferred to another tube, and 1 mL of isopropyl alcohol (Sigma-Aldrich Co., St. Louis, MO, USA) was added. This solution was incubated for 30 min at 4 °C and centrifuged for 8 min at 13,200×g at 4 °C. The pellet was mixed with 500 μL of 75% ethanol and centrifuged for 10 min at 13,200×g at 4 °C. The supernatant was discarded, and the pellet was dried in room air for 5 min. The pellet was dissolved in 50 μL of diethylpyrocarbonate (DEPC, Sigma-Aldrich Co.) water and stored at −75 °C. Electrophoresis using an agarose gel and ethidium bromide staining was performed to confirm the RNA quality and integrity.

### Reverse transcriptase- polymerase chain reaction (RT-PCR)

Total RNA was extracted from the urothelium and detrusor muscle tissues of rat bladders of the PBOO group and controls using TRIzol reagent and the chloroform method. Complementary DNA was synthesized using the First Strand cDNA Synthesis Kit (Fermentas Inc., Glen Burnie, MD, USA) with oligo-dT as the primer. Primers F-5′-CCCGCCGGCTATTGGTGACTT-3′ and R-5′-ATGACCACCCCGGCT CGTATGT-3′ were used to amplify NRG1 (358-bp). ErbB2 (483-bp) cDNA was synthesized using F-5′-AGCCCCCAGCCC GAGTATGTGAAC-3′ and R-5′-GAGCCGTCTGCCCTGGAT-GTAATG-3′. Oligomers F-5′-TCTCGAGCCAGCAAGAGCAGGAT-3′ and R-5′-GCCAGCAGAATAGCCAAGATT-3′ were used to synthesize cDNA of the M2 muscarinic receptor (557-bp). Primers F-5′-GATGGCATGGACTGTGGTCA-3′ and R-5′-GTCATCATCTCCGCCCCTTC-3′ were used to produce cDNA of glyceraldehyde 3–phosphate dehydrogenase (GAPDH, 189-bp). PCR reactions were performed and programmed in three steps as follows: 94 °C for 5 min, 35 cycles of 94 °C for 30 s/58 °C for 40 s/72 °C for 30 s, and 72 °C for 5 min. PCR product sizes were analysed using electrophoresis on 2% agarose gels. GAPDH was used as a housekeeping gene.

### Protein extraction and western blotting

Frozen tissue of the urothelium and detrusor muscle obtained from cystectomy was ground with a mortar and pestle and homogenized at 4 °C in 1× radioimmunoprecipitation assay buffer (Sigma-Aldrich®, St. Louis, MO, USA, ready-to-use solution containing 150 mM of NaCl, 1.0% IGEPAL® CA-630, 0.5% sodium deoxycholate, 0.1% SDS, 50 mM Tris, pH 8.0.). Homogenates were transferred to Eppendorf tubes on ice, incubated for 5 min and centrifuged at 13,000 g for 20 min at 4 °C. The supernatant was transferred to newly labelled Eppendorf tubes, and total protein content was measured using a Bradford assay kit (Thermo scientific, Pittsburgh, PA, USA), according to the manufacturer’s instructions. The samples were mixed with lysis buffer, boiled at 95 °C for 5 min, and cooled. A sample containing 50 μg of total protein was loaded onto a 12% sodium dodecyl sulphate polyacrylamide gel, and proteins were separated using electrophoresis for 2 h. Proteins were transferred to polyvinylidene fluoride membranes (Dyneon, 3 M, St. Paul, MN, USA) using a trans-blot semidry transfer cell (Mini-Protean® TetraCell, Bio-Rad, Hercules, CA, USA) for 1 h at 15 V. The membrane was washed with Tris-buffered saline Tween-20 and incubated with 5% skimmed milk for 1 h to block non-specific binding. Membranes were incubated with the following primary antibodies overnight at 4 °C: NRG1 monoclonal mouse antibody (1:200, Abcam, Cambridge, MA, USA), ErbB2 polyclonal rabbit antibody (1:200, Abcam), and M2 muscarinic receptor polyclonal goat antibody (1:200, Santa Cruz Biotechnology, Inc., Dallas, TX, USA). Membranes were washed with PBS 3 times, and the antibody binding was detected using goat anti-mouse antibodies for NRG1 (1:200, Abcam), goat anti-rabbit antibodies for ErbB2 (1:200, Abcam) and donkey anti-goat antibodies for M2 muscarinic receptor (1:2000, Santa Cruz Biotechnology) conjugated to horseradish peroxidase. The secondary antibodies were incubated at room temperature for 1 h. Bands were visualized using enhanced chemiluminescence (Vilber Lourmat, Marne La Vallée, France).

### Statistical analysis

Data were analysed using the Mann-Whitney test for comparisons between two groups. A *p*-value <0.05 was considered statistically significant. All data are presented as the means ± standard deviation. All calculations were performed using IBM SPSS Statistics ver. 20.0 (IBM Co., Armonk, NY, USA).

## Results

### Western blot assay of NRG1, ErbB2 and M2 muscarinic receptors

**Fig. 1:**
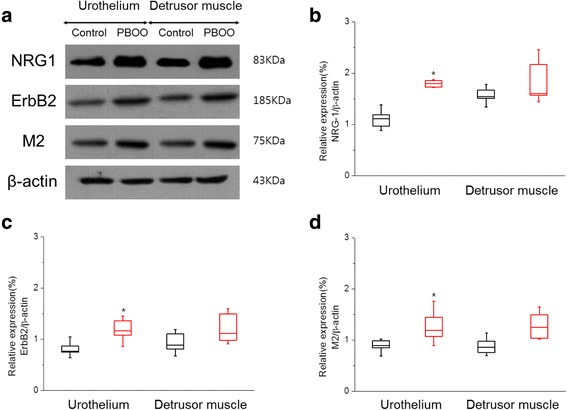
Representative immunoblots (**a**) and comparison of relative expression of NRG-1 (**b**), ErbB2 (**c**) and M2 muscarinic receptor (**d**) protein expression in urothelium and detrusor muscle between control (black plot) and PBOO group (red plot). β-actin was used as the loading control. Values of NRG-1, ErbB2 and M2 muscarinic receptor expression are normalized relative to β-actin expression. The asterisk indicates a significant difference (Mann Whitney test *p* < 0.05)

We investigated the expression of M2 muscarinic receptor (75 kDa), NRG1 (44 kDa) and ErbB2 (185 kDa) receptor using western blot analysis in the urothelium and detrusor muscle of bladders from PBOO-induced OAB and control rats (Fig. 1a). The relative intensities and distributions of NRG1, the ErbB2 receptor and M2 muscarinic receptor in the urothelium were approximately 1.6-, 1.5-, and 1.4-fold higher, respectively, in the PBOO group than the control group (1.80 ± 0.15 vs. 1.10 ± 0.15, 1.24 ± 0.28 vs. 0.82 ± 0.15, and 1.25 ± 0.25 vs. 0.90 ± 0.11, *P* < 0.05) (Fig. 1b, c, d). The relative intensities and distributions of NRG1, the ErbB2 receptor and M2 muscarinic receptor in detrusor muscle were approximately, 1.2-, 1.3-, and 1.4- fold higher, respectively, in the PBOO group than the control group (1.86 ± 0.36 vs. 1.60 ± 0.17, 1.23 ± 0.27 vs. 0.93 ± 0.18, and 1.20 ± 0.30 vs. 0.89 ± 0.15, *P* > 0.05) (Fig. 1b, c, d).

### mRNA expression of NRG1, ErbB2 receptor, and M2 muscarinic receptor

**Fig. 2:**
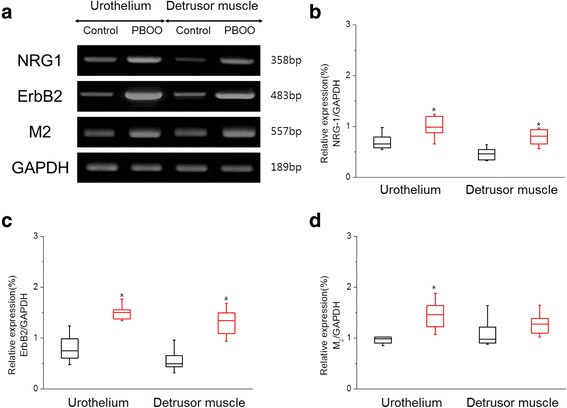
Representative RT-PCR expression (**a**) and comparison of relative expression of NRG-1 (**b**), ErbB2 (**c**) and M2 muscarinic receptor (**d**) mRNA expression in urothelium and detrusor muscle between control (black plot) and PBOO group (red plot). GAPDH was used as the loading control. Values of NRG-1, ErbB2 and M2 muscarinic receptor expression are normalized relative to GAPDH expression. The asterisk indicates a significant difference (Mann Whitney test *p* < 0.05)

We evaluated the mRNA expression levels of NRG1 (358 bp), ErbB2 (483 bp) and M2 muscarinic receptor (557 bp) in the urothelium and detrusor muscle of bladders from PBOO-induced OAB and control groups using RT-PCR. The mRNA expression level of NRG1 was higher in the urothelium and detrusor muscle of rat bladders with PBOO-induced OAB than control rats (Fig. 2a, b) (*P* < 0.05). The mRNA expression of ErbB2 exhibited a similar pattern to NRG1; however, the PBOO group exhibited significantly higher levels of ErbB2 in the urothelium and detrusor muscle (Fig. 2a, c) (*P* < 0.05). There was no significant difference in the mRNA expression level of M2 muscarinic receptors in the detrusor muscle of rat bladder (*P* > 0.05). A significant difference was observed in the mRNA expression levels of M2 muscarinic receptors in the urothelium of rat bladder between the PBOO group and control (Fig. 2a, d) (*P* < 0.05).

## Discussion

BOO produces significant alterations in bladder structure, such as detrusor hypertrophy, and function, including elevated detrusor pressure during contraction and detrusor instability, which lead to impairment in the storage and emptying of the bladder and morphological and physiological transformations of the urothelium, suburothelium and detrusor muscle [17, 18]. The urothelium affects nerve activity, contraction of the detrusor muscle and bladder function by evoking the release of various mediators in response to the sensing of mechanical and chemical stimuli [6]. A recent human study demonstrated urothelium dysfunction, increased suburothelial inflammation and apoptosis, and altered sensory protein expression in the mucosa of bladders in patients with BOO [18].

Muscarinic receptors may be an important factor in the voiding reflex of bladders. Activation of muscarinic receptors is one of the mechanisms driving detrusor muscle contractions. Five different subtypes of muscarinic receptor are expressed in the urothelium of mouse bladder [19], and the M2 muscarinic receptor favourably couples to the inhibition of adenylyl cyclase via the Gi subunit [20]. The density of M2 muscarinic receptors is greater than the density of M3 muscarinic receptors in the bladder and other smooth muscles, but subtype-selective muscarinic receptor antagonists demonstrated that M3 muscarinic receptors exhibit higher affinity than M2 muscarinic receptors under normal bladder conditions [21]. An increase in total and M2 muscarinic receptor density is observed in certain pathological conditions, especially in hypertrophied bladders following spinal cord injury and denervation. The pathophysiology of BOO-induced OAB includes mediation of bladder afferent neural transduction via overexpression of M2 and M3 muscarinic, P2X3 [22] and TRPV4 [23] receptors in the urothelium. However, the expression of M2 and M3 muscarinic receptors in BOO-induced urothelium is controversial. Bschleipfer [24] et al. reported that BOO did not induce significant alterations in the human urothelial non-neuronal cholinergic system at the mRNA level. However, Jiang [18] et al. noted that patients with BOO exhibited increased P2X3 and M2 muscarinic receptor expression but lower M3 muscarinic receptor expression in the bladder mucosa. Braverman and Ruggieri [25] reported that rat bladder hypertrophy induced by BOO, independent of bladder denervation, shifted the muscarinic receptor subtype mediating bladder contraction from M3 towards M2 muscarinic receptors. Kim [22] et al. reported overexpression of M2 and M3 muscarinic and P2X3 receptors in the urothelium of the BOO group, and overexpression of M3 muscarinic receptors in the muscle layer of the BOO group was also noted. However, our study demonstrated no significant difference in the immunoreactivity of M2 muscarinic receptors in the detrusor muscle, but the PBOO group exhibited increased M2 muscarinic receptor expression in the urothelium. This evidence supports the significant roles of M2 muscarinic receptors in BOO-induced bladder dysfunction.

There are few reports on NRG1 in the urinary bladder, but research on NRG1 in the heart is very active. NRG1 is a member of the EGF family, and NRG1/ErbB signalling may function in many tissues to create, and perhaps repair, tissue architecture via accommodation of cell-to-cell interactions in some organs, such as breast and heart [26]. Okoshi et al. demonstrated that NRG1 produced a negative inotropic effect via activation of the muscarinic response in cultured cardiomyocytes [27]. Activation of muscarinic receptors may lead to improved fractional shortening via modulation of the inotropic response to beta-adrenergic stimulation [28]. Another role of NRG1 in the heart was regenerative activity following focal ischaemia. Bersell [14] et al. demonstrated that NRG1/ErbB4 signalling induced mononucleated cardiomyocytes to proliferate, which resulted in the cardiac repair mechanisms in old myocardial infarction without influencing the level of apoptosis. NRG1/ErbB signalling is likely an important paracrine mediator of cell-to-cell interactions that exerts a negative inotropic effect via activation of the muscarinic response and helps regenerate cardiomyocytes following cardiac ischaemia via an NRG1-mediated ErbB2/ErbB4 signalling pathway.

Yamaguchi [29] et al. reported that BOO-produced bladder ischaemia resulted in urothelial damage via activation of hypoxia-inducible factor pathways and further oxidative stress and denervation disturbed urothelial homeostasis. They suggested that the extent of bladder dysfunction in chronic bladder ischaemia depended on the duration and degree of ischaemia. A moderate degree of bladder ischaemia may result in detrusor overactivity and storage symptoms via a post-junctional hypersensitivity because of partial denervation of the detrusor muscle and sensitization of afferent pathways. The progression from moderate to severe bladder ischaemia increases damage to the detrusor muscle and the progression of denervation, which may result in detrusor underactivity and voiding symptoms. The results of the present study demonstrated increased protein and mRNA expression levels of NRG1/ErbB2 in the urothelium, but not the muscle layer. Our findings suggest that upregulated NRG1/ErbB receptors may exert a functional role via receptors located in the urothelium as a result of muscarinic receptor activation and NRG1/ErbB may be associated with detrusor smooth muscle proliferation against chronic bladder ischaemia. Arrighi [30] et al. reported that M2 and M3 muscarinic receptors mediated human detrusor smooth muscle contraction and proliferation via Akt/PI3K and MAP kinases. We previously reported the effects of NRG1 on the expression of nicotinic acetylcholine receptors via the ErbB2/ErbB3-PI3K-MAPK signalling cascade in major pelvic ganglion [31]. Upregulated NRG1 may play a role in detrusor muscle proliferation using the PI3K-MAPK signalling cascade via upregulated M2 muscarinic receptors.

## Conclusions

We found remarkable changes in muscarinic and NRG1/ErbB receptors in PBOO-induced OAB. These results suggest that the overexpression of muscarinic and NRG1/ErbB2 receptors in the bladder, especially the urothelium, play a potential role in the afferent sensory responses of the urinary bladder. The NRG1 may be up-regulated by activation of the muscarinic receptors through PI3K/MAPK signaling cascades which is 10 multiple binding profiles to affect the ErbB2 receptor and protects the bladder against bladder ischemia by BOO. However, comprehension about muscarinic and neuregulin/ErbB receptors is limited, and further research about agonists, antagonists and knock-out mice should be performed. The results of these studies would increase our understanding of the development OAB induced by BOO and contribute to improved treatments for overactive bladder.

## Abbreviations

BOO: Bladder outlet obstruction
BPH: Benign prostatic hyperplasia
OAB: Overactive bladder
PBOO: Partial BOO
